# Immunogenic cell death by neoadjuvant oxaliplatin and radiation protects against metastatic failure in high-risk rectal cancer

**DOI:** 10.1007/s00262-019-02458-x

**Published:** 2019-12-31

**Authors:** Simer J. Bains, Hanna Abrahamsson, Kjersti Flatmark, Svein Dueland, Knut H. Hole, Therese Seierstad, Kathrine Røe Redalen, Sebastian Meltzer, Anne Hansen Ree

**Affiliations:** 1 Department of Oncology, Akershus University Hospital, P.O. Box 1000, 1478 Lørenskog, Norway; 2 Institute of Clinical Medicine, University of Oslo, Oslo, Norway; 3 Department of Tumor Biology, Oslo University Hospital, Oslo, Norway; 4 Department of Gastroenterological Surgery, Oslo University Hospital, Oslo, Norway; 5 Department of Oncology, Oslo University Hospital, Oslo, Norway; 6 Department of Radiology, Oslo University Hospital, Oslo, Norway; 7 Division of Radiology and Nuclear Medicine, Department of Research and Development, Oslo University Hospital, Oslo, Norway; 8 Department of Physics, Norwegian University of Science and Technology, Trondheim, Norway

**Keywords:** Immunogenic cell death, Rectal cancer, Oxaliplatin, Radiotherapy, Metastasis

## Abstract

**Objective:**

High rates of systemic failure in locally advanced rectal cancer call for a rational use of conventional therapies to foster tumor-defeating immunity.

**Methods:**

We analyzed the high-mobility group box-1 (HMGB1) protein, a measure of immunogenic cell death (ICD), in plasma sampled from 50 patients at the time of diagnosis and following 4 weeks of induction chemotherapy and 5 weeks of sequential chemoradiotherapy, both neoadjuvant modalities containing oxaliplatin. The patients had the residual tumor resected and were followed for long-term outcome.

**Results:**

Patients who met the main study end point—freedom from distant recurrence—showed a significant rise in HMGB1 during the induction chemotherapy and consolidation over the chemoradiotherapy. The higher the ICD increase, the lower was the metastatic failure risk (hazard ratio 0.26, 95% confidence interval 0.11–0.62, *P *= 0.002). However, patients who received the full-planned oxaliplatin dose of the chemoradiotherapy regimen had poorer metastasis-free survival (*P *= 0.020) than those who had the oxaliplatin dose reduced to avert breach of the radiation delivery, which is critical to maintain efficient tumor cell kill and in the present case, probably also protected the ongoing radiation-dependent ICD response from systemic oxaliplatin toxicity.

**Conclusion:**

The findings indicated that full-dose induction oxaliplatin followed by an adapted oxaliplatin dose that was compliant with full-intensity radiation caused induction and maintenance of ICD and as a result, durable disease-free outcome for a patient population prone to metastatic progression.

## Introduction

The potential to use the immune system to fight progressing cancer has opened new therapeutic avenues. Tumor-defeating immunity depends on both tumor-antigen recognition and the action of cytotoxic T cells, but is counterbalanced by tumor-induced immune tolerance. The latter can be edited by cancer immune therapies that revoke the evading T cell cytotoxicity. So far, this concept has proven successful in the treatment of a limited number of immunogenic tumors. Less immunogenic cancers, such as the majority of colorectal cancer (CRC) cases, will need additional stimulation to breach the immune tolerance in order for patients to achieve beneficial therapeutic responses [1]. Within this frame of reference, immunogenic cell death (ICD) implies the cytotoxic damage of tumor cells by either radiation or certain systemic remedies and the resulting priming of tumor-targeting T cells [2].

In CRC, the current standard-of-care therapies may induce ICD that invokes and maintains antitumor immunity. Specifically, emerging preclinical and clinical evidence supports the notion of oxaliplatin as an ICD-inducing agent [3–6]. The extracellular release of the high-mobility group box-1 (HMGB1) protein by the dying tumor cells, which facilitates cross-presentation of shed tumor antigens by dendritic cells to activate tumor-specific cytotoxic T cells, is an integral mechanism of the oxaliplatin-induced ICD [3, 7, 8]. In a similar fashion, ionizing radiation as a cytotoxic agent also provokes these responses, which at least theoretically may unleash systemic antitumor effects [9, 10] that eradicate occult or clinically established tumor manifestations at sites away from the radiation target volume (the abscopal effect [11, 12]).

The standard-of-care for patients with locally advanced rectal cancer (LARC) consists of neoadjuvant long-course chemoradiotherapy (CRT), containing a non-cytotoxic radiosensitizing dose of a fluoropyrimidine, followed by resection of the residual tumor tissue. This strategy has led to significantly improved local recurrence rates [13], but still as many as 30–40% of patients experience distant metastasis [14–16]. The addition of postoperative systemic therapy in this setting has not been convincing [16, 17]. Efforts have been made to improve LARC outcome by the use of neoadjuvant chemotherapy (NACT) prior to or immediately following the radiation [18–24].

In our prospective LARC study (NCT00278694), patients received 2 cycles (over 4 weeks) of the oxaliplatin-based Nordic FLOX regimen as induction NACT and sequential CRT with concomitant oxaliplatin weekly (over 5 weeks) with the aim to deliver additional systemic therapy in the neoadjuvant setting and intensify local radiation effects [25]. The study may have led to an ICD conceptual discovery in the high-risk patient population with 5-year progression-free survival (almost all events were metastatic progression) and overall survival (OS) that were remarkably good [25]. Patients who responded to the induction NACT by a pronounced rise in soluble immune factors that remained elevated during the sequential CRT, had significantly better progression-free survival than patients without such responses [26, 27], indicating that an advantageous systemic immune response had been invoked during the oxaliplatin-based neoadjuvant treatment.

In the current derivative study, we hypothesized that HMGB1 might be retrieved in the patients’ circulation as a direct measure of the ability of the cytotoxic agents to induce ICD over the neoadjuvant treatment course, essentially translating into durable disease-free outcome in a LARC population given curative-intent therapy, yet prone to metastatic progression.

## Materials and methods

### Patients

Eligible patients had histologically verified rectal adenocarcinoma that was considered high risk by magnetic resonance imaging (MRI): T2 cases that presented tumor threatening the anal levator muscles, T3 cases that had mesorectal fascia margin of less than 3 mm, T4 cases (organ-infiltrating tumor), or cases that had involved pelvic cavity lymph nodes (N1-2 disease). The full eligibility criteria and evaluation and follow-up procedures have been detailed previously [25]. The present study subpopulation of 50 patients was selected because of the completeness of biobank plasma samples and clinical data.

### Study design, treatment, and end points

This single-arm study was conducted to evaluate the safety and efficacy of the intensified neoadjuvant therapy [27]; the exploratory end points of this report were encouraged by findings from analysis of the safety and efficacy data. The induction NACT was given as 2 cycles of the Nordic FLOX regimen (oxaliplatin 85 mg/m^2^ on day 1 and bolus fluorouracil 500 mg/m^2^ and folinic acid 100 mg on days 1 and 2 every second week). The sequential CRT consisted of radiation delivered 5 days per week to a total dose of 50 Gy in 25 fractions to the tumor bed and 48 Gy in 23 fractions to regional lymph nodes, based on 2-/3-dimensional conformal planning models, with concomitant weekly oxaliplatin 50 mg/m^2^ and capecitabine 825 mg/m^2^ twice daily on days of radiotherapy. The neoadjuvant schedule was continuously adjusted according to treatment toxicity from each of the therapeutic agents, as detailed previously [27], principally by reducing the oxaliplatin dose scheduling to avoid compromising radiation delivery, as protraction of the total CRT time theoretically might permit tumor cell repopulation and thereby the survival and dissemination of therapy-resistant cell clones. Radical excision of the residual tumor was planned within 8 weeks after completion of the neoadjuvant treatment. Patients did not proceed to further therapy. Routine blood samples were collected within the standard patient follow-up. Tumor mutational *KRAS* status was determined for 39 (78%) of the present cases [28]. In addition to the routine diagnostic MRI performed at baseline and 4 weeks after CRT completion, 42 cases (84%) were also assessed after completion of NACT (post-NACT). The tumor boundary was manually contoured by the study radiologist on all tumor-containing axial *T*_2_-weighted images [29] at baseline and post-NACT. Tumor volume (*V*) change was calculated as ∆*V*_NACT_ = [(*V*_NACT_ − *V*_baseline_)/*V*_baseline_] × 100. The histologic scoring of the resected tumor specimens (ypTN stage) was recorded as treatment surrogate end point. One patient had disease progression in the pelvic cavity during the neoadjuvant treatment and, therefore, proceeded to palliative surgery; hence, histologic tumor response was not available. The patients included in this report were enrolled from 5 October 2005 through 2 December 2009 and final censoring was done on 8 August 2013 for recording of distant metastasis-free survival (DMFS) and OS.

### Measurement of plasma HMGB1

Patients had plasma sampled over 9 weeks: at baseline, post-NACT, and following CRT completion (post-CRT). The samples were stored at − 80 °C until analysis undertaken in April 2017 (i.e., after 86–138 months of storage). HMGB1 was analyzed with the Human HMGB1® ELISA Kit (Shinto, Kanagawa, Japan), following the manufacturer’s manual, after 1:2 sample dilution and using the mean value of two technical replicates for further analyses. The post-NACT plasma sample was missing for one patient, and this case was accordingly left out from some analyses.

### Statistical analysis

The HMGB1 absolute values were ln-transformed to achieve normality in estimate analyses and expressed as mean ± SEM. Associations between HMGB1 and various patient and disease factors were determined by independent samples *t*-test, one-way ANOVA, and Pearson correlation test, as appropriate. Alterations in HMGB1 during the neoadjuvant treatment were analyzed by paired samples *t*-test and described by profile plots with mean ± SEM. DMFS was calculated from the time of study enrollment to the day of metastatic progression, death of any cause, or end of follow-up (a maximum of 5 years after the date of surgery or at final censoring), whichever occurred first. OS was measured from the date of enrollment to death of any cause or final censoring. Associations between HMGB1 and DMFS and OS were analyzed with univariable and multivariable Cox proportional hazards models, and results were presented as hazard ratio (HR) with 95% confidence interval (CI). Due to the limited number of patients and events, potentially predictive factors other than age and sex were omitted from the multivariable models. The Kaplan–Meier method was used to estimate DMFS differences among groups with various CRT oxaliplatin doses, with the log-rank test to examine any statistical significance. All tests were two-sided and *P*-values less than 0.05 were considered statistically significant. Analyses were carried out using STATA version 15. GraphPad Prism version 7.0 was used for illustrations.

## Results

### Patient characteristics and circulating HMGB1

**Fig. 1:**
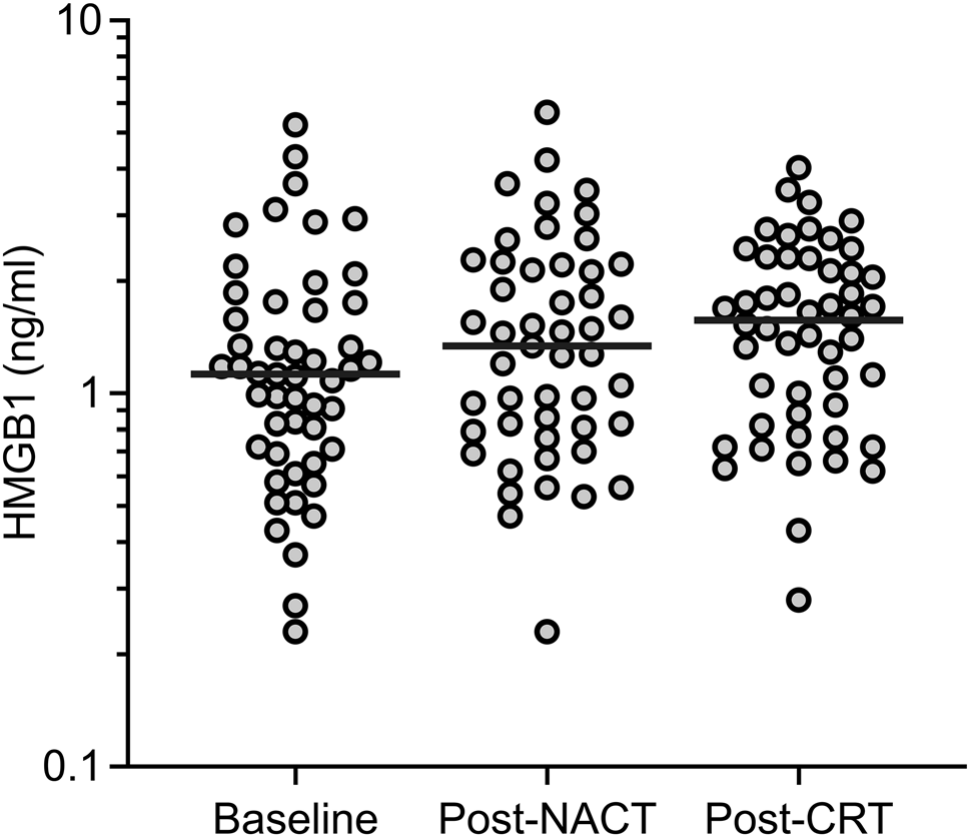
Plasma HMGB1 levels during NACT and sequential CRT. The horizontal line in each data cluster represents the median value

The 21 women and 29 men with median age of 56.5 years (range 30–73) presented with T2 (10%), T3 (58%), or T4 (32%) disease, the majority (82%) with involved lymph nodes and tumor wild-type *KRAS* status (67% of the 39 analyzed cases; Table 1). As illustrated in Fig. 1, the median plasma HMGB1 showed a modest increase from the absolute value of 1.13 ng/ml (range 0.23–5.25) at baseline through 1.34 ng/ml (range 0.23–5.67) post-NACT and 1.57 ng/ml (range 0.28–4.03) post-CRT, yet with a significant group difference between the baseline and post-CRT values of ln 0.23 ± 0.10 ng/ml (*P *= 0.029). Baseline HMGB1 was not associated with patient or tumor characteristics (Table 1).

**Table 1:** Baseline characteristics of the cohort

	*N* (%)	HMGB1 (ln mean ± SEM), ng/ml	*R*	P*
Median age (range), years	56.5 (30–73)		0.15	0.29
Sex				
Male	29 (58)	0.08 ± 0.13		
Female	21 (42)	0.15 ± 0.14		0.69
Tumor *KRAS* status				
Wild-type	26 (52)	0.10 ± 0.13		
Mutant	13 (26)	0.29 ± 0.23		
Unknown	11 (22)	–0.09 ± 0.17		0.39
T stage				
2	5 (10)	0.43 ± 0.18		
3	29 (58)	0.09 ± 0.15		
4	16 (32)	0.05 ± 0.12		0.54
N stage				
0	8 (16)	–0.03 ± 0.27		
1	5 (10)	0.27 ± 0.33		
2	36 (72)	0.12 ± 0.11		0.75
Median tumor volume (range), cm^3^	16.8 (1.1–135)		0.03	0.86

*By Pearson correlation test or independent sample *t*-test

### HMGB1 over the neoadjuvant treatment course

**Fig. 2:**
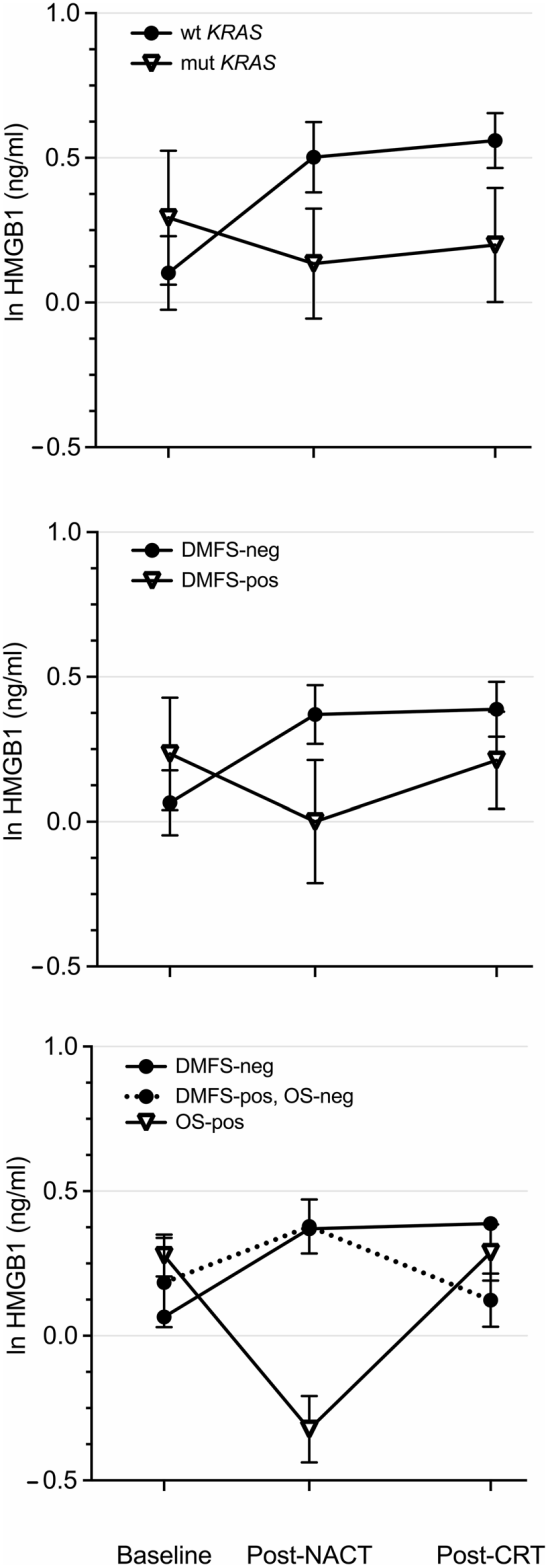
Plasma HMGB1 profile plots over the course of NACT and sequential CRT for various patient categories. Wild-type or mutant tumor *KRAS* status (the upper panel). Negative or positive for a DMFS event at study censoring (the middle panel). Negative or positive for an OS event at study censoring. The dashed profile represents cases alive with metastatic disease at censoring (the lower panel). *mut* mutant, *neg* negative, *pos* positive, *wt* wild-type

Analysis of tumor gene expression data sets has shown that mutant *KRAS* status is associated with reduced infiltration of cytotoxic T cells and suppression of the adaptive IFN-γ response in CRC [30], and a possible mechanism for mutant *KRAS*-driven CRC immune tolerance was recently reported [31]. In this context, the modest neoadjuvant HMGB1 alterations within the entire patient group segregated into distinct patterns when patients were categorized into those harboring tumors with mutant (*N *= 13) or wild-type (*N *= 26) *KRAS* status (the upper panel of Fig. 2). The wild-type group revealed a rise of ln 0.40 ± 0.13 ng/ml (*P *= 0.006) from baseline to NACT completion, whereas the mutant group had a slight decline. During the succeeding CRT, plasma HMGB1 did not essentially change in either of the groups.

Similarly, HMGB1 profile plots over the neoadjuvant treatment course were compared for patients who over the total follow-up period did (*N *= 13) or did not (*N *= 37) experience a DMFS event (the middle panel of Fig. 2). The group with the aimed study outcome (durable freedom from distant recurrence) had an increase in HMGB1 of ln 0.27 ± 0.12 ng/ml (*P *= 0.036) during NACT, after which the level was consolidated. In contrast, the patient group with metastatic failure had a non-significant decline initially before plasma HMGB1 reverted during the sequential CRT.

For OS (the lower panel of Fig. 2), the small group of patients who died during the follow-up period (*N *= 7) had a neoadjuvant HMGB1 profile pattern mirroring that of a positive DMFS event, with decline during NACT before reverting during CRT, both alterations at the margin of statistical significance (*P *= 0.053 and *P *= 0.049, respectively). Likewise, individuals alive at censoring (*N *= 43) displayed a pattern closely resembling that of patients without DMFS event. Acknowledging the small number when specifically examining those alive with metastatic disease at censoring (*N *= 6), it was still notable that the post-NACT HMGB1 was not maintained during the sequential CRT. Besides those pointed at, none of the described alterations within a group was statistically significant.

### HMGB1 and disease outcome

Since the plasma HMGB1 profiles suggested that alterations during NACT in particular (termed ∆HMGB1) might be indicative of disease outcome, we investigated whether ∆HMGB1 might correlate with early treatment effects (Table 2). This factor correlated moderately well with the change at the same time point of monocyte count (*N *= 46, *R *= 0.30, *P *= 0.040), indicating that an early systemic immune response might have been invoked. However, ∆HMGB1 was not associated with ∆*V*_NACT_ (the initial tumor volume response, *N *= 42) or ypTN response of the surgical specimen (*N *= 47–48), implying that a biological connection between ICD from the induction NACT and MRI-assessed and histologic tumor effects did not exist.

**Table 2:** ∆HMGB1 and correlations with disease and patient outcome factors

	*N* (%)	∆HMGB1 (ln mean ± SEM), ng/ml	*R*	P*
Thrombocytes^a^	49 (98)		0.21	0.16
Lymphocytes^a^	47 (94)		0.12	0.43
Neutrophils^a^	49 (98)		0.069	0.63
Monocytes^a^	46 (92)		0.30	0.040
Lactate dehydrogenase^a^	48 (96)		0.035	0.81
Erythrocyte sedimentation rate^a^	42 (84)		–0.010	0.95
Albumin^a^	49 (98)		–0.058	0.69
∆*V*_NACT_	42 (84)		0.014	0.93
ypT stage				
0–2	28 (56)	0.21 ± 0.15		
3–4	19 (38)	0.07 ± 0.17		0.53
ypN stage				
0	36 (72)	0.21 ± 0.13		
1–2	12 (24)	–0.02 ± 0.21		0.37
DMFS				
No event	36 (72)	0.27 ± 0.12		
Event	13 (26)	–0.23 ± 0.20		0.041
OS				
No event	42 (84)	0.26 ± 0.11		
Event	7 (14)	–0.60 ± 0.25		0.005
OS among cases with DMFS event^b^				
No event	6 (12)	0.19 ± 0.25		
Event	7 (14)	–0.60 ± 0.25		0.048

*By Pearson correlation test or independent sample *t*-test

^a^Alteration during the neoadjuvant chemotherapy

^b^Cases with DMFS event were analyzed for OS

At censoring, the median time to a DMFS event was 11.0 months (range 3.3–26.4) with 31.4 months (range 8.0–52.6) to death for the deceased (all from the metastatic disease), while the median follow-up time for participants still alive was 77.3 months (range 45.0–94.4). No difference in DMFS or OS was observed between the patient groups with mutant or wild-type *KRAS* tumor status (not shown). As may be expected from the profile plots, ∆HMGB1 distinguished between patient groups with and without DMFS and OS events (*P *= 0.041 and *P *= 0.005, respectively) and metastatic disease leading to death or not (*P *= 0.048; Table 2). Moreover, as shown by Table 3, the higher the ∆HMGB1, the lower were the risks of metastatic failure and death. Age and sex, which might have affected the long-term outcome, were included in the Cox regression models, but ∆HMGB1 remained an independent predictor of DMFS (HR 0.26, 95% CI 0.11–0.62, *P *= 0.002) and OS (HR 0.14, 95% CI 0.04–0.51, *P *= 0.003); other prognostic factors (e.g, the status of extramural venous invasion at baseline MRI or involved lymph nodes in the resected specimen) were omitted in the multivariable models. Post-estimation tests of the proportional hazard assumption were not significant, supporting the validity of the models.

**Table 3:** ∆HMGB1 and patient factors in prediction of outcome

	Univariable			Multivariable		
	HR	95% CI	*P*^a^	HR	95% CI	P*
DMFS						
∆HMGB1	0.46	0.23–0.92	0.028	0.26	0.11–0.62	0.002
Age	0.94	0.89–0.98	0.010	0.90	0.84–0.95	< 0.001
Sex^a^	1.25	0.41–3.83	0.693	2.42	0.73–8.09	0.15
OS						
∆HMGB1	0.25	0.09–0.70	0.008	0.14	0.04–0.51	0.003
Age	0.96	0.90–1.03	0.234	0.91	0.83–0.99	0.024
Sex^a^	0.96	0.22–4.30	0.960	1.42	0.31–6.51	0.65

*By Cox proportional hazards models; all univariable covariates included in the multivariable model

^a^Female as reference

### Oxaliplatin during CRT and disease outcome

**Fig. 3:**
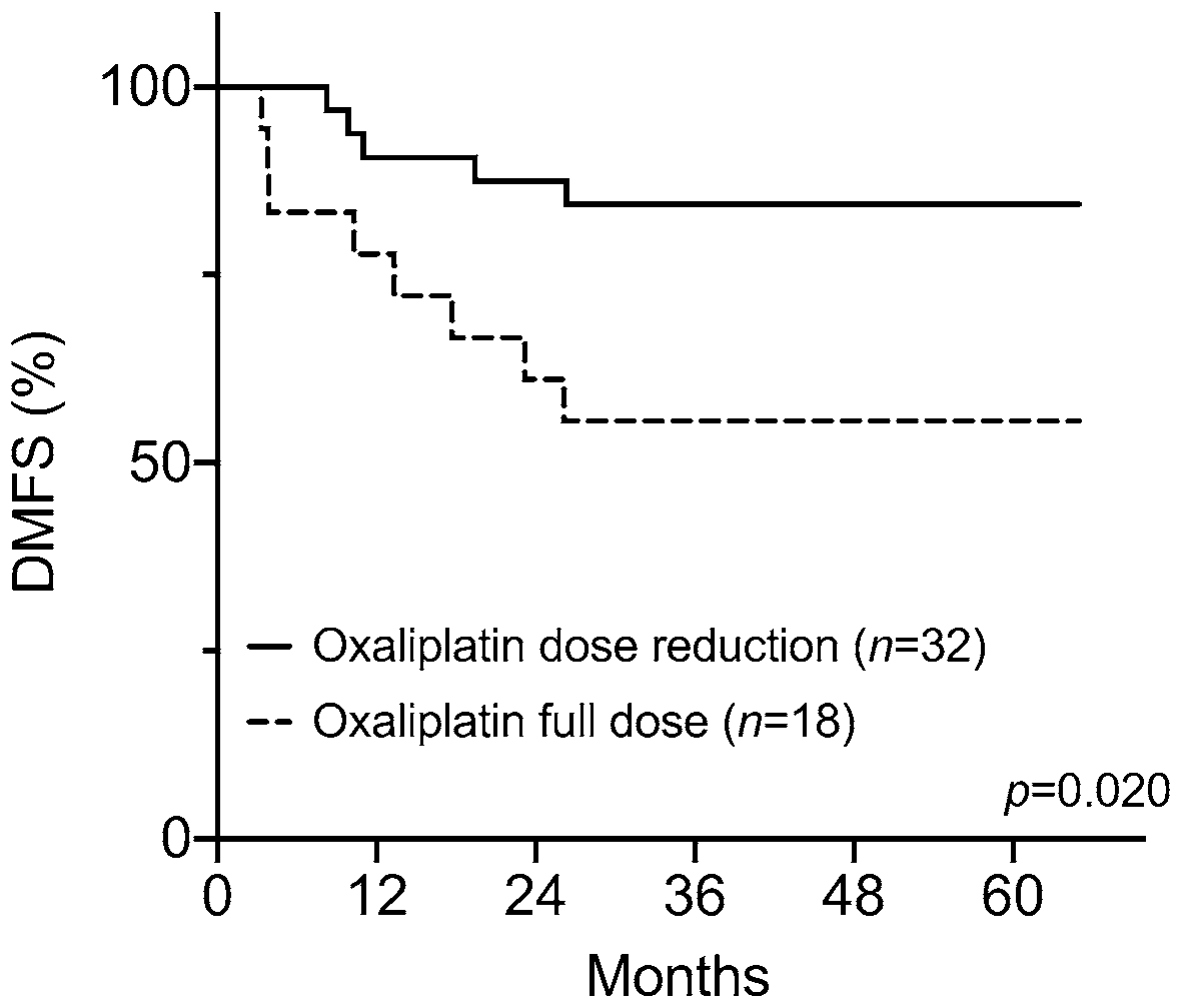
DMFS for patients receiving the full-planned or reduced oxaliplatin dose during the neoadjuvant CRT

To tentatively clarify whether the ICD induction during NACT would be indicative of the disease outcome in its own capacity, the patients’ treatment status during the sequential CRT was specifically examined. The study subjects were first categorized into those who during CRT received the total oxaliplatin dose that was planned in the protocol (90–100%, *N *= 18) and those who had a modest reduction (70–89% of the planned dose, *N *= 8) or a substantial reduction (< 70% of the planned dose, *N *= 24) because of treatment toxicity. Whereas none of patients (0 of 8) with the modest oxaliplatin dose reduction experienced a DMFS event, 21% of individuals (5 of 24) with a substantial reduction did; of note, 44% of patients (8 of 18) who received the full-planned oxaliplatin dose later had metastatic failure. Hence, the last-mentioned group had considerably worse outcome compared to patients who had the oxaliplatin dose reduced during CRT (*P *= 0.020; Fig. 3). With respect to the radiotherapy, all of the 50 cases in the present analysis received the total dose of 50 Gy without interruption in radiation delivery, which is critical for the elimination of clonogenic cells within the source of disease dissemination (the radiation target volume).

## Discussion

High-risk LARC patients who during oxaliplatin-based induction NACT experienced rise in circulating HMGB1, regarded as a measure of ICD induction, remained free of metastatic failure following the curative-intent multimodal therapy if certain conditions, probably interconnected, prevailed over the course of the sequential CRT. One was that oxaliplatin was reduced from the protocol-planned dose as result of toxicity, which averted breach of the radiation delivery (i.e., maintained the cytotoxic treatment effect locally), and another was that the plasma HMGB1 remained elevated. Altogether, this suggests that a durable disease-free outcome for this patient population prone to metastatic progression was contingent on ICD invoked by short-course oxaliplatin during NACT, resulting in tumor-defeating immune activity that required protection from systemic toxicity (i.e., oxaliplatin below a cumulative cytotoxic dose) during CRT. Our findings in this clinical setting are consistent with experimental studies reporting that oxaliplatin causes ICD and HMGB1 release [3, 8, 32, 33] and the possible abscopal effect of radiotherapy [11, 12].

For the entire group of 50 patients, plasma HMGB1 showed a modest variation over the neoadjuvant treatment course, which first led us to investigate whether tumor mutational *KRAS* status might identify cases with ICD response. Not unexpectedly given recent experimental data [30, 31], patients harboring wild-type *KRAS* tumors displayed a significant rise in HMGB1 during the induction NACT; while in clear contrast, patients harboring mutant *KRAS* tumors were without the initial oxaliplatin-induced ICD response. These observations suggest that mutant *KRAS* tumors may become less immunogenic by cytotoxic therapy than their wild-type counterparts, which may further contribute to the more aggressive metastatic behavior of the mutant entity [34]. However, we found no survival differences between the mutant and wild-type *KRAS* groups in this primarily non-metastatic LARC setting, which might have been a chance finding due to the limited number of patients with known tumor *KRAS* status. Analyses of larger LARC populations have shown that mutant *KRAS* tumors had poorer local response to neoadjuvant therapy, but survival data were not reported [35, 36]. We did not observe any correlation between therapy-induced ICD and the local tumor response. Altogether, the observations argue that wild-type *KRAS* status is a contributory and not a causative factor for tumor ICD.

Patients who met the study’s main end point—freedom from distant recurrence following oxaliplatin-containing systemic and radiation-based therapies before definitive surgery—had an initial increase in circulating HMGB1 before consolidation over the remaining neoadjuvant treatment. Specifically, the ICD induction during NACT was a strong predictor of DMFS and OS—the higher the ∆HMGB1, the lower the risk of metastatic failure and death (all recorded deaths were from metastatic disease). On the contrary, the patient group that later experienced DMFS events showed a non-significant decline in plasma HMGB1 during NACT before reverting. Regarding the OS outcome and acknowledging the small numbers, it was still notable that, firstly, individuals alive with metastases at censoring seemed to have had a reduction in HMGB1 during CRT following an initial rise, and secondly, patients who later died had a decline in HMGB1 already during NACT. The subsequent increase during CRT for the latter group is consistent with the notion that radiation causes ICD [37]; however, in this high-risk population, it did not by itself protect against poor outcome. In summary, declining plasma HMGB1 at any stage of the neoadjuvant treatment was unfavorable for the long-term outcome. Whether a deficient ICD induction may relate to established risk factors for LARC outcome, such as tumor invasion into rectal extramural veins or pelvic lymph node metastases surviving the neoadjuvant therapy, is unknown.

Consolidation of the NACT-induced HMGB1 during CRT seemed to be required for a favorable DMFS, which led us to investigate how tumor-defeating immune activity might have been maintained. All patients received the total radiation dose without interruption in delivery, likely upholding cytotoxic effects on clonogenic tumor cells within the source of disease dissemination. Because capecitabine dose adjustment in CRT was not associated with long-term outcome in this LARC study [27], we examined the impact of oxaliplatin dosing in the current analysis. Patients treated in accordance with the planned oxaliplatin dose intensity during CRT had significantly poorer DMFS than those who had oxaliplatin dose reduction because of toxicity. Our interpretation of these observations is that oxaliplatin at a continuous cytotoxic dose during CRT might have quenched the tumor-targeting T cells that had been activated during NACT and maintained by the ongoing radiation-dependent ICD. As a result, this abated an ongoing immune response that otherwise would enable eradication of occult microscopic tumor at distant sites (the abscopal effect of CRT) in patients prone to develop metastatic disease. In practical terms, patients who tolerated full oxaliplatin doses throughout the entire neoadjuvant therapy had oxaliplatin-resistant tumor or normal tissues or both. In a large LARC study, patients randomized to concomitant oxaliplatin had significantly improved disease-free survival compared to those in the standard CRT arm [16]. In this particular trial, the oxaliplatin dose (50 mg/m^2^ weekly in 4 of 5 radiotherapy weeks, thus corresponding to the modest reduction category in our study) secured patient compliance to the study protocol [16]. Other randomized studies in the same setting used higher cumulative doses of oxaliplatin in the CRT regimen [38–40] and, therefore, did not provide any indications as to whether it might have acted as an ICD-inducing agent.

Of note, HMGB1 and the monocyte count changed in parallel during NACT. HMGB1 stimulates tumor antigen-presenting dendritic cells, which arise together with monocytes within the common myeloid progenitor lineage [41], to cause cytotoxic T-cell activation [7, 42]. These responses are among the main mechanisms for oxaliplatin effects [3, 7, 8]. On the other hand, we found no correlations between ∆HMGB1 and treatment effects on the local disease, such as tumor response to the induction NACT at MRI (∆*V*_NACT_) or histologic response in the resected tumor specimens (ypTN stage), the latter a commonly used surrogate end point for neoadjuvant therapy. Altogether, these findings support the notion that ICD, rather than the conventional tumor responses, may represent a fundamental oxaliplatin effect of consequence for the survival end points.

This report has intrinsic shortcomings. The analyses were neither preplanned nor prespecified in the original statistical analysis plan, but encouraged by emerging evidence in recent years and along the conduct of this hypothesis-generating study. Furthermore, the cohort was relatively small and the study was single-armed. On the other hand, the overall results appeared to be robust and statistically significant, clinically plausible and relevant, and in line with previous studies. Yet, circulating HMGB1 is an isolated surrogate marker for complex ICD mechanisms, and supportive analyses should be included in future ICD studies. One example is the possible measurements of factors involved in tumor DNA-evoked immunogenicity, such as cytosolic DNA species that behave as immune response signals [43] regulated by therapeutic radiation [44, 45].

In summary, this study provides evidence that full-dose induction oxaliplatin followed by an adapted oxaliplatin dose that is compliant with full-intensity radiation results in induction and maintenance of ICD. In neoadjuvant treatment of high-risk LARC, this may translate into long-term survival without metastatic progression. Tumor wild-type *KRAS* status seems to be a contributory factor in the ICD generation. When the optimum dosing and timing of administration are known, conventional chemotherapy and radiotherapy may be combined with cancer immune therapy in a rational manner to further improve outcome.

## Abbreviations

CI: Confidence interval
CRC: Colorectal cancer
CRT: Chemoradiotherapy
DMFS: Distant metastasis-free survival
FLOX: The Nordic FLOX regimen (oxaliplatin, fluorouracil, folinic acid)
HMGB1: High-mobility group box-1
HR: Hazard ratio
ICD: Immunogenic cell death
LARC: Locally advanced rectal cancer
MRI: Magnetic resonance imaging
NACT: Neoadjuvant chemotherapy
OS: Overall survival
